# High-performance hole conductor-free perovskite solar cell using a carbon nanotube counter electrode†

**DOI:** 10.1039/d0ra05975g

**Published:** 2020-09-30

**Authors:** Mustafa K. A. Mohammed

**Affiliations:** Technical Engineering College, Middle Technical University Baghdad Iraq mustafa_kareem97@yahoo.com

## Abstract

Carbon-based perovskite solar cells (C-PSCs) are the most promising photovoltaic (PV) due to their low material and manufacturing cost and superior long-term stability. This work compares the performance between gold (Au) and multi-wall carbon nanotube (MWCNT) electrodes for hole transport material (HTM)-free PSCs. Based on the obtained results, C-PSCs showed remarkable power conversion efficiency (PCE) and negligible hysteresis. Indeed, under optimized conditions, MWCNTs demonstrated superior performance as a counter electrode (CE) for HTM-free PSCs, leading to a PCE of 15.56%, which is comparable to the current state-of-the-art materials. Also, the presence of MWCNTs in the cell architecture enhances the collection and injection of holes at the perovskite/MWCNT interface and as a result, improves the external quantum efficiency (EQE) and current density because the recombination process is quenched. This improvement is confirmed by impedance spectroscopy (EIS), photoluminescence (PL), current/voltage (*J*–*V*), and EQE measurements. Moreover, MWCNTs could act as a protective layer and enhance the PSC stability. C-PSC was more stable than that of traditional PSC based on Au, which could maintain 80% of its primary PCE for long-periods of storage in moist conditions.

## Abstract 2

Carbon-based perovskite solar cells (C-PSCs) are the most promising photovoltaic (PV) due to their low material and manufacturing cost and superior long-term stability.

## Graphical abstract

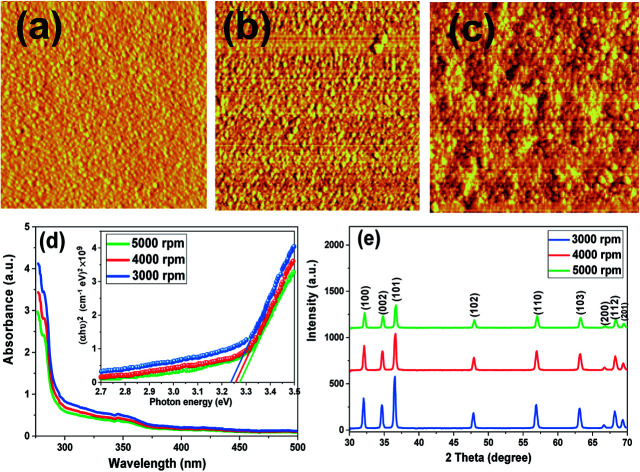

## 1. Introduction

Currently, the traditional PVs that dominate the solar industry, silicon solar cells, have difficulties to meet the commercialization requirement for low-cost devices because of their high raw material price and complicated industrialization process.^1–3^ PSCs, a promising technology for power conversion, have quickly improved and become a notable subject of discussion in the field of solar cells because of the prominent optical and electrical features of the organo-metal trihalide perovskite materials like CH_3_NH_3_PbX_3_ (X = Cl, Br, I) that have a greater light absorption coefficient, higher charge diffusion length, ambipolar carrier transport ability, and adjustable bandgap.^4–6^ Interestingly, the PCE of perovskite photovoltaics has boosted rapidly from 3% to 25% by optimizing the production procedure and device architecture.^7^ Improving the preparation process and PCEs would make perovskite-based photovoltaic competitive in future manufacturing.^8^

In the typical form of PSCs, methylammonium lead iodide (MAPI_3_) is used as a light-harvesting layer. This material is sandwiched between the electron transport material (ETM) and HTM.^9^ However, it is noteworthy that all PSC devices employ noble metals as the CE in tandem with the HTM working as the electron blocking material, leading to high-costs and instability issues.^10^ The HTM is highly expensive at present and would restrict its use in many applications. Besides, the vacuum deposition technique used for the metal-based CE is also highly energy-consuming. Undoubtedly, it is worth fabricating HTM-free PSCs and substituting the metal-based CE with low-cost and readily obtainable substances.^11^

The first HTM-free PSCs with a PCE of 5.5% were presented by Etgar *et al.*, revealing that the MAPI_3_ perovskite could act as the HTM and an absorber simultaneously, simplifying the photovoltaic structure, decreasing its price and enhancing the stability of PSCs.^12^ Nevertheless, this configuration and approach are an encouraging strategy; however, several disadvantages still prevent its application. Moreover, the hysteresis effect is observed in HTM-free PSCs based on Au electrodes, thus restricting their future application.^13–15^ In the past few years, carbon substances such as carbon black, bio-carbon, bulk and ultrathin graphite, and carbon nanotubes, with an energy alignment comparable to that of gold, have been employed in carbon-based PSCs.^16–19^

Previously, Ku *et al.* reported the fabrication of fully printable processed mesoscopic perovskite/titanium dioxide (TiO_2_) HTM-free PSCs with carbon CE. Employing a common drop-coating method to infiltrate the perovskite into three mesoporous materials showed a device PCE of 6.65%.^20^ In 2015, Zonghao *et al.* used carbon black/graphite as the CE and demonstrated that the perovskite light-harvester produced immediately within the titanium dioxide (TiO_2_)/nickel oxide (NiO)/carbon structure achieved a PCE of 11.4% under AM1.5G illumination.^21^ Anyi *et al.* fabricated C-PSCs *via* employing a double film of TiO_2_ and zirconium dioxide (ZrO_2_) penetrated with perovskite. The PSCs exhibited a PCE of 12.8%.^22^ Bulk and ultrathin graphite CEs have been applied in PSCs by Miao *et al.* to facilitate hole extraction from the perovskite to the CE. Correspondingly, the efficiency of the cell increased from 12.64% to 14.08% by replacing bulk graphite with ultrathin graphite.^18^ Recently, Pei *et al.* used carbon film as the low-temperature CE in printable mesoscopic PSCs and the highest PCE of 14.04% was accomplished.^23^ In 2020, Liguo *et al.* reported ultra-low-price bio-carbon materials for HTM-free PSCs and showed a high PCE of 12.8%.^17^

TiO_2_ is usually employed as the ETM in perovskite-based photovoltaics. Nevertheless, using TiO_2_ as a hole-blocking layer in PSCs may be an obstacle toward the commercialization of photovoltaics as a result of an elevated temperature sintering process (400–500 °C), and the lower charge mobility in titanium dioxide causes an unbalanced charge injection and disintegration of PSC performance.^24^ Therefore, an alternative ETM such as zinc oxide (ZnO) that has a wide bandgap and excellent charge mobility has been examined.^25^ ZnO-based PSCs need a lower sintering temperature (80–120 °C) than the TiO_2_-based PSCs.^26^ Unfortunately, the direct contact between the perovskite and ZnO causes decomposition of the perovskite crystals on the surface of the ZnO layers on account of the existence of hydroxide groups, which suppresses the PCE of ZnO-based PSCs.^27–29^ To solve this problem, a buffer layer between the ZnO and perovskite layers has been used to reduce the degradation of the perovskite layer. A buffer layer can efficiently separate ZnO and the perovskite, which allows large crystal to grow with thermal annealing.^30^

This article reports the fabrication of HTM-free PSCs with the structure of FTO/ZnO/PEI/perovskite/Au based on Au and ZnO as the CE and electron-blocking material, respectively. The impact of spinning speed on the structural, optical, and morphological properties of ZnO films was investigated and demonstrated to be a significant parameter that influences the PSC performance. The HTM-free PSCs fabricated at a spinning speed of 5000 rpm revealed a highest PCE of 12.81% with open circuit voltage (*V*_oc_) of 0.84 V, short circuit current (*J*_sc_) of 23.2 mA cm^−2^, and fill factor (FF) of 65%. To further improve the HTM-free PSC performance, MWCNTs were used as the CE for ZnO-based PSCs. Interestingly, the PCE of the device was boosted from 12.81% to 15.56% with less hysteresis after replacing the Au with a MWCNT electrode.

## 2. Experimental

### 2.1. Synthesis of ZnO

The ZnO nano-powder (NP) was prepared through a cheap and facile precipitation route.^31^ A one-step method with high-scale production and excellent purity is desirable for the cost effective synthesis of ZnO-NPs (more details in the ESI†).

### 2.2. Fabrication of FTO/ZnO/PEI/PVK/Au device

Fluorine-doped tin oxide (FTO, 15 Ω sq^−1^, Solaronix) coated-glass was employed as a substrate. Zinc powder (98%, Merck) and hydrochloric acid (3 M, Sigma-Aldrich) were used to etch the FTO substrates. Then, each substrate was washed under ultra-sonication in a diluted detergent, ethanol, and isopropanol (99%, Merck) for 30 minutes and rinsed with distilled water, dried at 200 °C for two hours, and treated with ultraviolet-ozone for 15 minutes. ZnO-NPs were dissolved in chloroform (CHCl_3_, 99%, Merck) and coated three times on the FTO glass at various speeds (3000, 4000, 5000 rpm) for 40 seconds and each depositing layer was sintered at 80 °C for 20 minutes. To synthesize the 15 nm buffer layer, poly-ethyleneimine (PEI, 24 000 g mol^−1^, Sigma-Aldrich) was dispersed in 1% of 2-methoxyethanol (CH_3_OCH_2_CH_2_OH, 99.8%, Merck), then spun at 4000 rpm for 20 s and sintered at 80 °C for 30 minutes. To grow the MAPI_3_ perovskite film on the surface of PEI buffer, 0.482 g of lead iodide (PbI_2_, 99%, Sigma Aldrich) powder was dispersed in dimethylformamide (DMF, 98%, Sigma-Aldrich) and dimethyl sulfoxide (DMSO, 99%, Sigma-Aldrich) with a volume ratio of 3 : 1 at 60 °C. Then, methylammonium iodide (MAI, Dyesol) was supplemented and stirred for 30 minutes. The mixture was spun at 3000 rpm for 30 seconds with added ethyl acetate (C_4_H_8_O_2_, Sigma-Aldrich), and then sintered at 100 °C for 10 minutes. Finally, to complete the device fabrication process, 80 nm of Au electrode was sputtered in a vacuum vessel with 10^−5^ mbar on the top of the MAPI_3_ film.

### 2.3. Fabrication of FTO/ZnO/PEI/PVK/MWCNTs device

The C-PSC architecture was fabricated by replacing the Au electrode with a MWCNT electrode according to the previous experiment for the fabrication of the FTO/ZnO/PEI/perovskite/Au device. MWCNT was obtained from http://www.cheaptubes.com, Grafton, USA with lengths of less than 40 μm and an outer diameter distribution over 8–15 nm (more details in the ESI†).

## 3. Results and discussion

### 3.1. Raw materials analysis

The MWCNT electrode, ZnO-NPs, and perovskite were characterized by a series of measurements, as shown in Fig. S1–S3 and Table S1 (more details in the ESI†).

### 3.2. ZnO thin films analysis

**Fig. 1:**
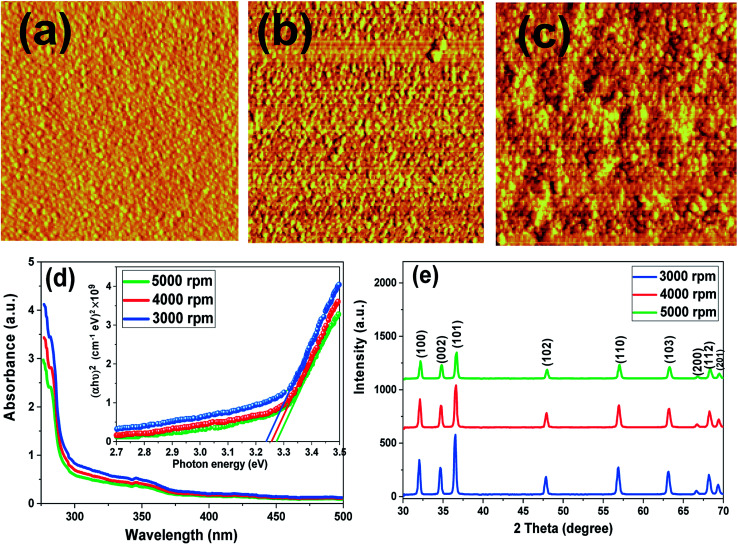
AFM for ZnO films spin-coated at (a) 5000 rpm, (b) 4000 rpm, and (c) 3000 rpm, (d) absorption spectra and Tauc plots (inset), (e) XRD patterns.

The dependence of the topography, particle size and homogeneity of the ZnO films on the spinning speed was observed by AFM imaging. The surface topography and grain boundary of the ZnO layers change with the spinning speed. As can be seen in Fig. 1a–c, the images demonstrate that the surface of the ZnO films consisting of many spherical particles uniformly spreads over the substrate with a crack-free and dense microstructure. The microstructure of the ZnO film prepared by a spinning speed of 5000 rpm reveals a high density of small particle sizes, which is typical when using a hole-blocking material in HTM-free PSCs. In Fig. 1b, by decreasing the speed to 4000 rpm, the ZnO film shows larger particles and a less dense surface, but very flat and smooth surfaces. The ZnO film deposited by the spinning speed of 3000 rpm (Fig. 1c) has a less uniform surface than the other two films and the size of the particles became larger.

Fig. 1d illustrates the absorption spectra of ZnO films spin-coated at different speeds. As the figure shows, at all spinning speeds, ZnO films are extremely transparent in the visible region, which meets the demands of photovoltaic applications well. Over all three films, the major peak is at about 345 nm but with a little shift in intensity.^9^ By comparing the three coated films, we observe that the ZnO spin-coating at 5000 rpm had less absorbance and, thus, more transparency than the other two films. According to the UV-vis plots and the FESEM images, the optical transmittance of the three films would decrease with an increase in their particle size during the coating process. Therefore, it can be assumed that as the film quality enhances, the transparency of the ZnO film increases.

As depicted in the inset of Fig. 1d, the bandgap of ZnO films were calculated using the Tauc plot.^32–34^ The magnitudes serve to support our particle size findings according to which a smaller particle size is expected to possess a higher bandgap (3.28 eV for the film coated at 5000 rpm) and a larger particle size is suggested to possess a lower bandgap (3.25 eV for the film coated at 4000 rpm and 3.24 eV for the film coated at 3000 rpm). The bandgap for ZnO films in this study decreases as compared to the bulk value (3.37 eV).^35^ In order to estimate the crystal structure of ZnO films deposited at different spinning speeds, XRD analysis was performed and presented in Fig. 1e. ZnO films deposited by a spin coating method seem to have polycrystalline structures and match well with the XRD pattern of ZnO-NPs.^36^ The intensities of the main peaks are reduced at higher spinning speed due to the elimination of more solution from the substrate that results in decreasing film thickness.

### 3.3. ZnO/PEI/perovskite heterojunction analysis

**Fig. 2:**
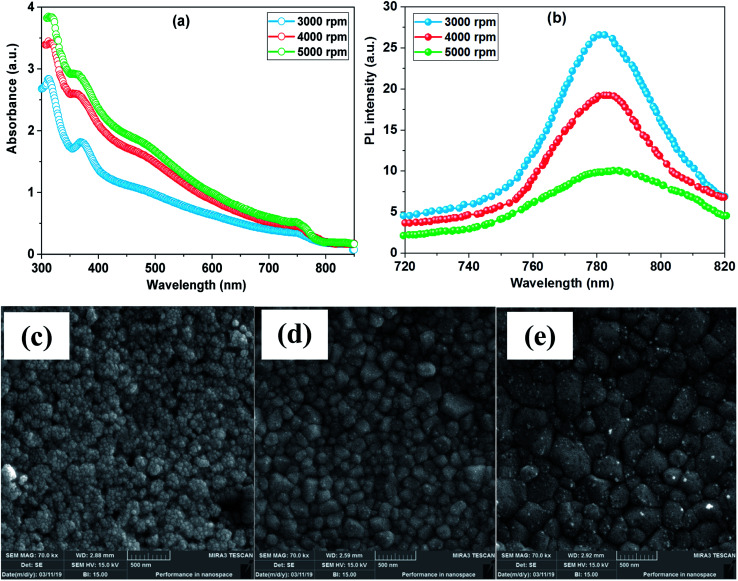
(a) absorption spectra, (b) PL spectra, and FESEM images for perovskite films coated on PEI/ZnO/FTO substrate at (c) 3000 rpm, (d) 4000 rpm, and (e) 5000 rpm.

Fig. 2a depicts the absorption spectra of FTO/ZnO/PEI/perovskite films for a set of samples fabricated at different spinning speeds of the ZnO layer. The plots show a higher intensity absorption spectrum for the perovskite film with spin-coated ZnO at 5000 rpm. The absorption spectra show a broad absorption band in the visible range with two peaks at 370 nm and 760 nm. The optical absorbance for perovskite film increases with the increasing spinning speed of the ZnO layer where the heterojunction coated at 5000 rpm has the highest absorbance, followed by 4000 rpm and 3000 rpm with the lowest absorbance. Thus, the enhanced optical absorbance was ascribed to an increase in perovskite material loading, high uniformity and better surface coverage of perovskite over the ZnO substrate at a higher spinning speed.

Furthermore, PL measurements were performed to study the impact of the ZnO layer spun at varying speeds on charge carrier transport and their corresponding plots are presented in Fig. 2b. An important PL intensity suppression is observed when perovskite layers are in contact with ZnO films at higher spinning speeds. The high suppression is due to the excited electron receiving the density of states and acting as an effective charge carrier by transporting from the perovskite harvesting layer, thus reducing the charge recombination rate.^37^

FESEM morphology images (Fig. 2c–e) prove that depositing the perovskite layer of spin-coated ZnO at high speed enlarged the grain size and no pinholes were present. From Fig. 2c, one can see that the perovskite film is grown with high number of invisible of grain boundaries with tiny pinholes. Compared to the PVK deposited on ZnO at 3000 rpm, the perovskite deposited on ZnO at 4000 rpm reveals visibly larger grain dimensions with highly dense morphology (Fig. 2d). Perovskite films where the precursor PbI_2_ was deposited on ZnO with an optimum spinning speed of 5000 rpm had a larger grain size (Fig. 2e). The small and smooth surface of ZnO films spun at 5000 rpm increased the solubility of lead iodide and improved its reaction with the MAI. This improved morphology film can boost charge extraction due to reduced charge recombination.

### 3.4. Device performance

**Fig. 3:**
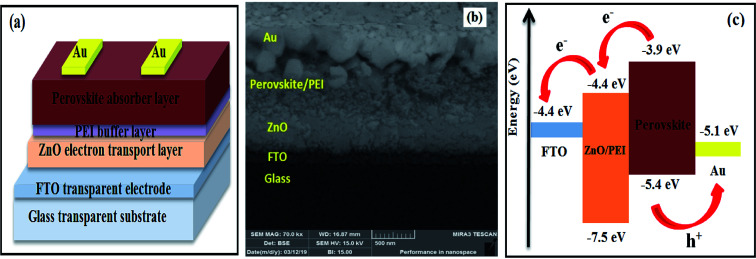
(a) Schematic illustration of the sandwich structure of the PSCs fabricated in this study: FTO front contact, ZnO ETM, PEI buffer layer, MAPI_3_ absorber layer and Au back contact, (b) cross-sectional FESEM image for HTM-free PSC, and (c) energy levels of the individual device components and possible electronics.

The spinning speed of the ZnO film was controlled at 3000 rpm, 4000 rpm, and 5000 rpm to fabricate PSCs labeled as device1, device2, and device3, respectively. Additionally, to compare the C-PSC with typical Au-PSC, cells were fabricated by using the MWCNT electrode and labeled as device4. Fig. 3a shows the device architecture of HTM-free PSCs fabricated directly on FTO substrates. The cross-section micrograph was visualized by FESEM and each layer was labeled, as presented in Fig. 3b. As has been previously reported in the literature, the better seamless interfacial contact, the faster charges can be transported. Obviously, the negligible interfacial gap between ZnO and the perovskite film could be observed in device3 based on the Au CE, which facilitated charges transferring from the perovskite to ZnO ETM and Au electrode. Fig. 3c illustrates the energy level graph of the films in the fabricated PSCs. It can be deduced from the figure that application of ZnO as an ETM matches the perovskite energy level. This promotes the electron transport from the absorber film, which is MAPI_3_ material here. In addition, due to its wide band gap, ZnO is an appropriate hole-blocking material matching the perovskite film. The −7.5 eV valence band in zinc oxide is much higher than the −5.4 eV valence band in the perovskite film, resulting in reduced charge recombination.

**Fig. 4:**
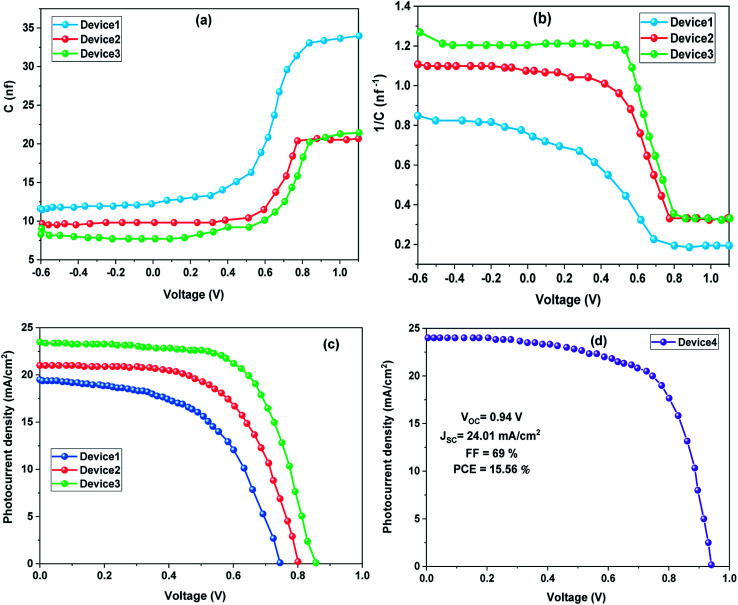
(a) *C*–*V* measurements of HTM-free PSCs based on Au electrodes, (b) Mott–Schottky plot, (c) *J*–*V* curves of HTM-free PSCs based on Au electrodes, and (d) *J*–*V* curve of HTM-free PSC based on MWCNT electrode.

Fig. 4a displays the change in junction capacitance with voltage for FTO/ZnO/PEI/perovskite/Au cells fabricated at different spinning speeds of the ZnO layer. From this figure, there is an increase in the capacitance with increasing voltage bias. This change in capacitance is caused by differences in the width of the depletion layer.^38^Fig. 4b reveals the relationship between the bias voltage and 1/*C*^2^ (Mott–Schottky plots). The built-in potential (*V*_bi_) can be determined by graphing the voltage with 1/*C*^2^ and identifying the intercept of the linear portion of the plot with a voltage-axis. We obtained 0.72 V, 0.83 V, and 0.9 V of *V*_bi_ from device1, device2, and device3, respectively. Due to many trapped states and ion migration in the perovskite layers or at the interfacial contact where ions and electrons are easily hindered at the interface, *V*_bi_ will decrease due to the screening effect if electrons are hindered at interface states and more ions are accumulated.^39^

To probe the performance of HTM-free PSCs, the *J*–*V* measurements of these devices were carried out under typical AM1.5G illumination (100 mW cm^−2^), as shown in Fig. 4c and their corresponding photovoltaic parameters are described in Table 1. The device3 based on the Au electrode demonstrated the optimum PCE of 12.81%, and the corresponding PCE of device2 and device1 was 10.41% and 7.87%, respectively. In general, the important boost in PCE is essentially assigned to the improved *V*_oc_ and FF. The improved open circuit voltage might be attributed to the improved charge transfer that can facilitate the collection of charges, which leads to an enhanced *V*_oc_. Simultaneously, as the spinning speed increases from 3000 to 5000 rpm, the *J*_sc_ increases from 19.3 mA cm^−2^ to 23.2 mA cm^−2^ and results in an increase in PCE from 7.87% to 12.81% (Fig. S4†). This improvement in photovoltaic parameters is related to the increased light harvesting because of an enlarged perovskite material loading in the ZnO/PEI, which provides a dense perovskite layer possessing better crystallinity.^20^ Also, this result ties in well with the steady-state PL findings, wherein the significant quenching of the PL of the MAPI_3_ perovskite indicates that charge extraction in this layer is enhanced.

**Table 1:** Performance of the champion HTM-free PSCs based on different ZnO ETMs

PSCs	*J*_sc_ (mA cm^−2^)	*V*_oc_ (V)	FF (%)	PCE (%)
Device1	19.3	0.74	56	7.87
Device2	21.0	0.8	62	10.41
Device3	23.2	0.85	65	12.81

When the MWCNT electrode is introduced into the HTM-free PSC (Fig. 4d), the device performance is significantly improved. For C-PSC, the *J*_sc_ is increased from 23.2 to 24.01 mA cm^−2^ and the FF is boosted from 65 to 69%, yielding a 15.56% PCE, suggesting contact is improved between the perovskite film and MWCNT CE. A more conductive CE results in improved charge collection and therefore increased FF, which in turn boosts the efficiency. This testing is comparable to those of other previous studies related to C-PSCs, as summarized in Table 2. These cells have a lower PCE under light illumination, except for the device with a MWCNT-doped perovskite film.^40^

**Table 2:** Performance comparison of HTM-free PSCs from previous reports based on carbon CEs

Device structure	Carbon type	PCE (%)	Ref.
FTO/c-TiO_2_/m-TiO_2_/PVK/C	Graphene	10.06	41
Mesoscopic FTO/c-TiO_2_/m-TiO_2_/ZrO_2_(PVK)^a^/C	Carbon	10.64	42
FTO/c-TiO_2_/m-TiO_2_/PVK/C	Coal	10.87	43
FTO/c-TiO_2_/m-TiO_2_/PVK/C	Candle soot	11.02	44
FTO/TiO_2_/NiO^b^(PVK)/C	CB^c^/graphite	11.4	21
Mesoscopic FTO/c-TiO_2_/m-TiO_2_/m-ZrO_2_(PVK)/C	CB/graphite + ink	11.8	45
FTO/c-TiO_2_/m-TiO_2_/PVK/C	MWCNT	12.6	19
Mesoscopic FTO/c-TiO_2_/m-TiO_2_/m-ZrO_2_(PVK)/C	Bulk graphite	12.63	18
Mesoscopic FTO/c-TiO_2_/m-TiO_2_/m-ZrO_2_(PVK)/C	Carbon	12.8	22
FTO/c-TiO_2_/m-TiO_2_/PVK/C	Bio-carbon	12.82	17
Mesoscopic FTO/c-TiO_2_/m-TiO_2_/m-ZrO_2_(PVK)/C	Ultrathin graphite	14.07	18
FTO/c-TiO_2_/TiO_2_-Al_2_O_3_^d^/PVK/C	SWCNT^e^-graphite/CB	14.7	46
ITO/SnO_2_^f^/PVK-MWCNT_0.5_/C	Carbon	16.25	40
FTO/ZnO/PEI/PVK/C	MWCNT	15.56	This work

a PVK: perovskite.

b NiO: nickel oxide.

c CB: carbon black.

d Al_2_O_3_: aluminum oxide.

e SWCNT: single wall carbon nanotubes.

f SnO_2_: tin oxide.

**Fig. 5:**
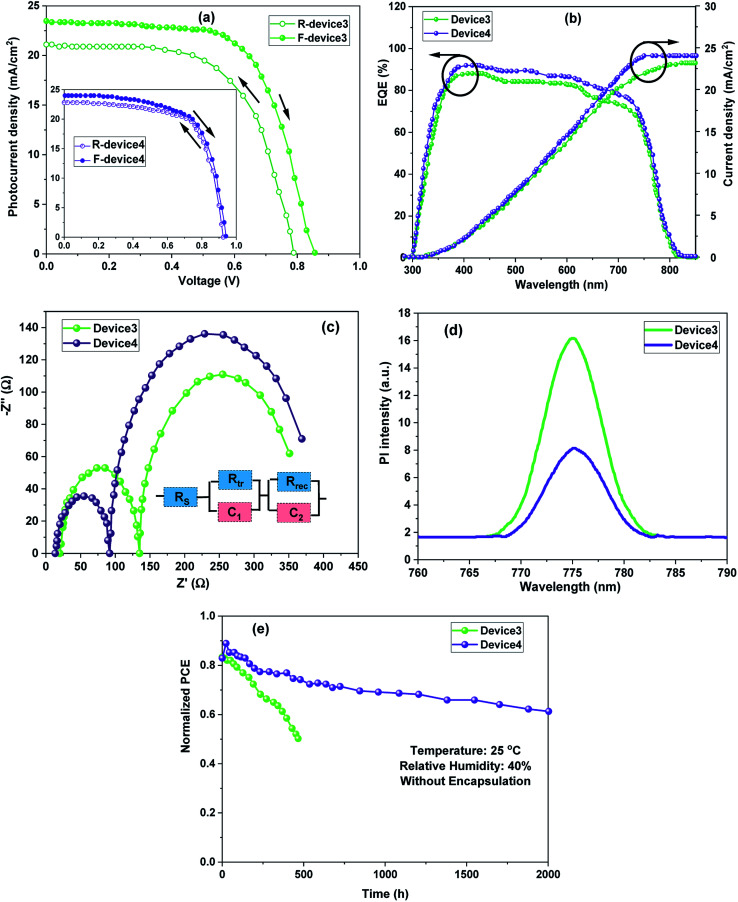
(a) Hysteresis effect, (b) EQE spectra, (c) Nyquist plot, (d) steady-state PL spectra, and (e)Stability tests of HTM-free PSCs based on MWCNT and conventional Au electrodes.

In order to compare the C-PSCs based on the MWCNT electrode with champion PSCs based on Au electrodes, several measurements such as the *J*–*V* hysteresis effect between forward (F, from *J*_sc_ to *V*_oc_) and reverse (R, from *V*_oc_ to *J*_sc_) scans, EQE, EIS, PL spectroscopy, and stability tests were accomplished (Fig. 5). As plotted in Fig. 5a, the Au-based PSCs demonstrated a significant hysteresis and distortion in the *J*–*V* plot. Such a hysteresis phenomenon convolutes the real *J*–*V* plot in the F-scan and causes overestimation in the R-scan. In contrast, it can be observed from the inset of Fig. 5a that the replacement of Au with MWCNT suppresses the hysteresis phenomenon of the device. It has been proposed that an irregular hysteresis in the photovoltaic scan can be ascribed to the carrier recombination at the perovskite/transferring layer interface. In our approach, the PSCs were fabricated without a HTM. Therefore, the hole transport depends on the perovskite/CE interface. Obviously, MWCNTs reduced the charge recombination in the C-PSC, which may have balanced the electron and hole transfer within the devices and this may contribute to the decreased *J*–*V* plot hysteresis. This finding is in good agreement with previous reports.^19^

To confirm the *J*_sc_ changes after using two kinds of CEs, the EQE spectra of the PSCs were recorded and are presented in Fig. 5b. The integrated photocurrent densities from EQE tests were 23.18 and 24.02 mA cm^−2^ for the cells based on Au and MWCNTs, respectively, which are in good agreement with the corresponding *J*_sc_ magnitudes measured from the *J*–*V* characteristics of the cells. Improvements in the EQE spectrum as compared to the conventional Au-PSC over the entire wavelength range for the C-PSC based MWCNT electrode were observed. We attribute the outstanding carrier transport/extraction properties to the presence of MWCNT electrode. However, when the MWCNT electrode is used for the HTM-free PSCs, their narrower band gap relative to Au, suitable energetic alignment, and high conductivity can efficiently collect the photo-generated charges. Such a dynamic strategy can result in a larger interface area of the perovskite/MWCNT leading to fewer grain boundaries and carrier recombination, hence promoting charge injection.

To gain further insights into the charge transport dynamics and *J*_sc_ enhancements, EIS and PL measurements were recorded. At high frequency, a small semicircle is related to the charge transport resistance (*R*_tr_), and a large semicircle at low frequency represents the carrier recombination resistance (*R*_rec_).^25^ As depicted in the Nyquist plot (Fig. 5c), a smaller *R*_tr_ and larger *R*_rec_ could be observed in the cells with MWCNT electrodes, which indicated that the charge collection and injection were effective and carrier recombination was reduced. This was consistent with the PL plots in Fig. 5d, where all of the photoluminescence spectra were collected under the same conditions except for various counter electrodes. The weaker intensities represented the faster hole collection leading to a lower recombination rate. The results revealed that the PSC based on MWCNT electrodes had faster hole collection and lower recombination than devices based on Au electrodes.

The long-term stability of devices based on MWCNTs and traditional Au configurations examined without any encapsulation were stored inside a dry air box (40% RH) at room temperature (20–30 °C). As presented in Fig. 5e, devices based on MWCNTs were more stable than devices with the typical structure (FTO/ZnO/perovskite/PVK/Au). It could maintain about 80% of the initial PCE over 2000 h of aging time for C-PSC. Au-PSC revealed a much lower stability under the same test conditions. It is now accepted that in a moist ambience, H_2_O molecules cause the degradation of perovskites and result in serious topographical changes. Such topographical properties are damaging to the direct charge transport between the perovskite and charge transport layers. The presence of MWCNT electrodes provides a better interface with the perovskite, and hence provides more hole transport pathways. Compared to the device with Au, these low cost and stable HTM-free PSCs with low temperature-processed MWCNTs are impressive PVs.

## 4. Conclusion

Herein, HTM-free PSCs based on ZnO ETM and Au electrodes have been simply fabricated *via* the spin coating method at various spinning speeds in ambient air. Then, the champion ZnO-based PSC performance was compared with the C-PSC. At a high spinning speed of 5000 rpm, the champion PCE (up to 12%) of HTM-free PSC was obtained. The results demonstrated that the device performance was significantly affected by the crystallinity, morphology, and optical transmittance of ZnO films. The charge transport dynamics demonstrated the lower recombination and faster charge transfer in HTM-free PSCs. The replacement of Au by MWCNTs with optimized conditions resulted in a remarkable improvement in the PCE, significantly suppressed the J-V hysteresis of the cell, and enhanced the stability for long-term storage in humid conditions. To date, by employing MWCNTs as the CE, a PCE of 15.5% was accomplished, which is one of the highest PCEs for HTM-free PSCs. The increased *J*_sc_ and *V*_oc_ values are the major factors for this PCE boost. This considerable improvement in the device parameters of the C-based PSC is chiefly assigned to the efficient hole collection because of the better band energy alignment at the perovskite/C interface. We expect that this work will make a significant impact in designing MWCNT-based CEs for high-performance solar cells. Future research on HTM-free PSCs based on MWCNTs should concentrate on the enhancement of FF by judiciously improving the crystallinity of the MAPI_3_ absorber layer.

## Conflicts of interest

The authors declare that they have no conflict of interest.

## Supplementary Material

RA-010-D0RA05975G-s001
