# Supplementary material for: High-performance hole conductor-free perovskite solar cell using a carbon nanotube counter electrode
Source: RSC Adv. 2020 Sep 30;10(59):35831–9. doi: 10.1039/d0ra05975g (PMC9056886; doi:10.1039/d0ra05975g)
Supplement: RA-010-D0RA05975G-s001 [file RA-010-D0RA05975G-s001.pdf]

Electronic Supplementary Material (ESI) for RSC Advances.
This journal is © The Royal Society of Chemistry 2020

High-performance hole conductor-free perovskite solar cell using carbon nanotubes counter electrode

Mustafa K. A. Mohammed*
Technical Engineering College, Middle Technical University, Baghdad, Iraq
*Corresponding author email: mustafa_kareem97@yahoo.com

1. Experimental part

1.1. Synthesis of ZnO

Briefly, 0.5gr of zinc acetate (Sigma-Aldrich) was dispersed in 100 ml of absolute ethanol (99.9%, Sasma). Then, 1gr of oxalic acid (98%, Sigma-Aldrich) was dispersed in 100 ml of absolute ethanol and carefully added into the zinc acetate solution. The mixture was stirred at the 50 °C for 2 hours, dried at the 90 °C for one day, and sintered at 450 °C for 3 hours to obtain ZnO-NPs.

1.2. Fabrication of FTO/ZnO/PEI/perovskite/MWCNTs Device

In brief, 0.5 gr of MWCNT was dissolved in 30 ml ethyl alcohol and sonicated in ultrasonic device for 20 minutes. This solution was magnetically stirred for 15 minutes. The solution was subsequently centrifuged for 10 minutes at 4000 rpm/minute to eliminate other carbonaceous impurities. The solution was dissolved in ethyl alcohol, in which 15.6 ml terpilenol inserted. The mixture was then treated via ultrasonic probe for 10 minutes, stirred for 5 minutes, and ball-milled for 10 hours. Then, 3 grethyecellulose was inserted into the mixture. Finally, the ethyl alcohol was discarded with rotary evaporation. The MWCNT paste was doctor-bladed to grow the MWCNT layer and hot-pressed onto the surface of perovskite at 80 °C under a pressure of 20 MPa for 3 minutes.

1.3. Characterization

The crystal nature of the MWCNT was investigated via X-ray diffraction spectrometry (XRD, Bruker, D8 advance) and Raman spectroscopy (Renishaw MicroRaman, λ= 514 nm). The absorption spectrum of the MWCNT was measured through an ultraviolet-visible spectrophotometer (Ocean Optics, USB4000 UV-VIS). The transmission electron microscope (TEM, JEOL, JEM-2100) was carried out to visualize the morphological properties of MWCNT. The structural properties of the ZnO-NPs were investigated via XRD and Fourier transform infrared spectroscopy (FTIR, Burker, KBr disc). While, their morphology and chemical compositions were

assessed by field emission electron microscopy (FESEM, TESCAN, Mira 3) and energy dispersive spectroscopy (EDS), respectively. The crystallinity and absorbance of perovskite film were studied via XRD and UV-vis spectrophotometer, respectively. Photoluminescence (PL) spectrum of PVK film was collected by a PL system (Agilent, Varian Cary Eclipse Fluorescence) with an excitation at 405 nm. The atomic force microscopy (AFM, CSPM-5000) was performed to visualize the perovskite topography. Capacitance/voltage(C-V) measurements of the cells were characterized by a FLUKE PM6306 (LRC meter, 1 MHz). Current density/voltage (J-V) characteristics of photovoltaics were monitored by a Keithley Model 2400 under simulated sun with intensity of 100 mW/cm^2. The cells were masked through an active area of 0.2 cm^2. The external quantum efficiency (EQE, BunkoukeikiCEP-1500) was employed to estimate charge generation and collection in the solar cells using xenon lamp (150 W, USHIO) as a source of monochromatic light. The electrochemical impedance spectroscopy was utilized to study the Nyquist plots through an electrochemical workstation (Zennium 400147) under light irradiation and 0.6 V voltage of AC perturbation varying from 100 mHz to 4 MHz.

2. Results discussion

2.1. MWCNT measurements

Two diffraction peaks at 25.6° and 43.5° were observed in the XRD pattern correspond to the (002) and (100) crystal planes of graphite-like materials (JCPDS code 01-0646) [1], as presented in Figure 1a. To further prove the crystallinity of MWCNT, Raman spectra has been recorded, as illustrated in Figure 1b, where D-band (1330 cm^{-1}) and G-band (1571 cm^{-1}) attributed to the sp^3 amorphous defects and the graphene hexagon structure plane, respectively [2].What is more, the absorption spectrum (Figure 1c) of MWCNT exhibits a high band at 231 nm due to the $\pi \rightarrow \pi^*$ transition and the shoulder band at about 298 nm ascribed to $n \rightarrow \pi^*$ transitions [3]. The HRTEM image (Figure 1d) demonstrated that tube-like morphology without obvious crystal defects or impurity could be found for MWCNT.

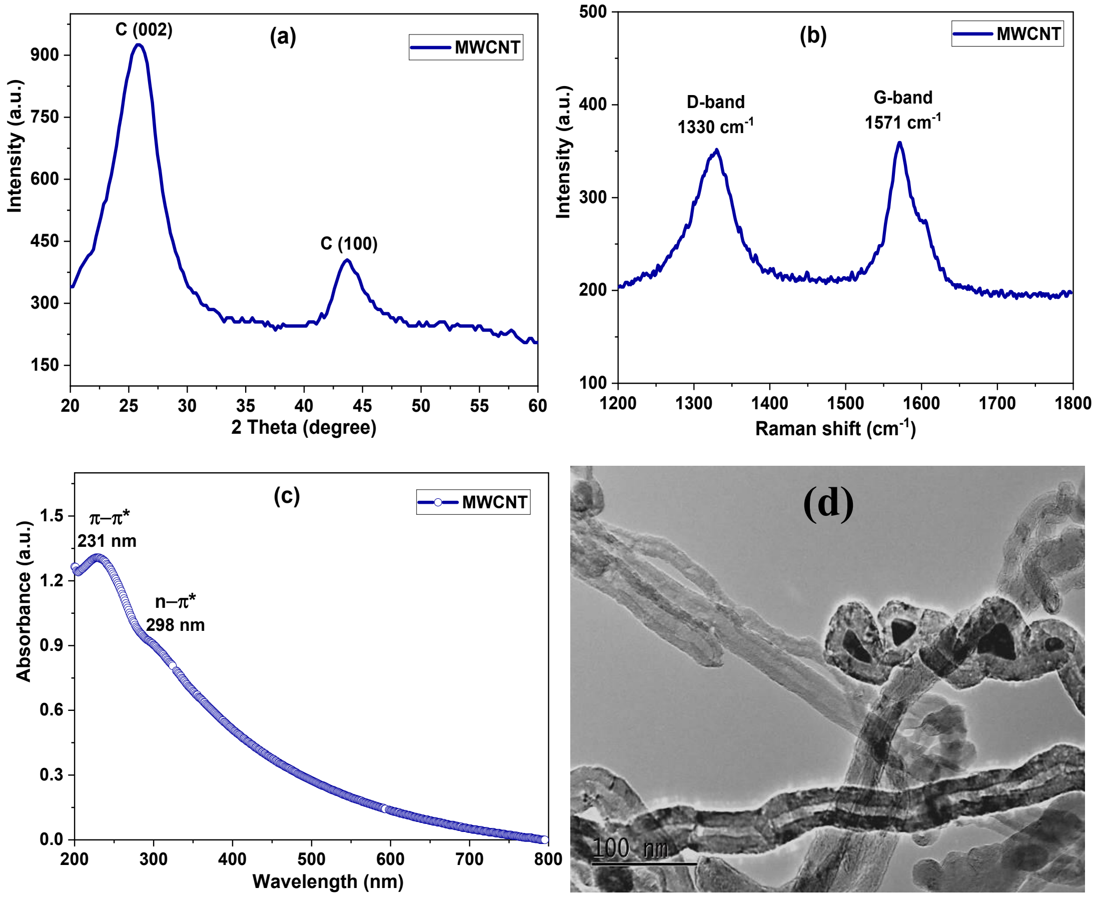

Figure 1 a) XRD pattern, b) Raman spectrum, c) absorption spectrum, and d) HRTEM image for MWCNTs.

2.2. Perovskite measurements

Figure 2a illustrates the XRD measurement of perovskite layer deposited using a spin coating process with optimized conditions. As shown in Figure 1a, the peaks at the two-theta values of 14.2°, 28.2°, 31.5°, and 43.9° attributed to the tetragonal microstructure of organic-inorganic perovskite are ascribed to (110), (220), (310), and (330) crystal planes of perovskite, respectively [4]. The peak at about 12.14° of PbI_2 was not observed in the XRD pattern of perovskite, indicating complete conversion of PbI_2 into perovskite. Thus the amount of PbI_2 residue was suppressed and the perovskite crystallinity was improved. The UV-vis spectrum of perovskite layer (Figure 2b) shows a broad absorption band towards the visible range with one significant peak at 760 nm. Moreover, according to the corresponding Tauc curves; band gap of perovskite film with is 1.55 eV as that of the standard material. The high optical absorbance and small band gap fulfills the requirements of absorber material well. The steady-state PL spectrum of spin-coated perovskite is presented in Figure

2c. A broad and intense peak is located at 785 nm corresponding to the perovskite material. AFM topography image (Figure 2d) indicates that the perovskite material deposited with uniform, highly dense and pinholes-free film.

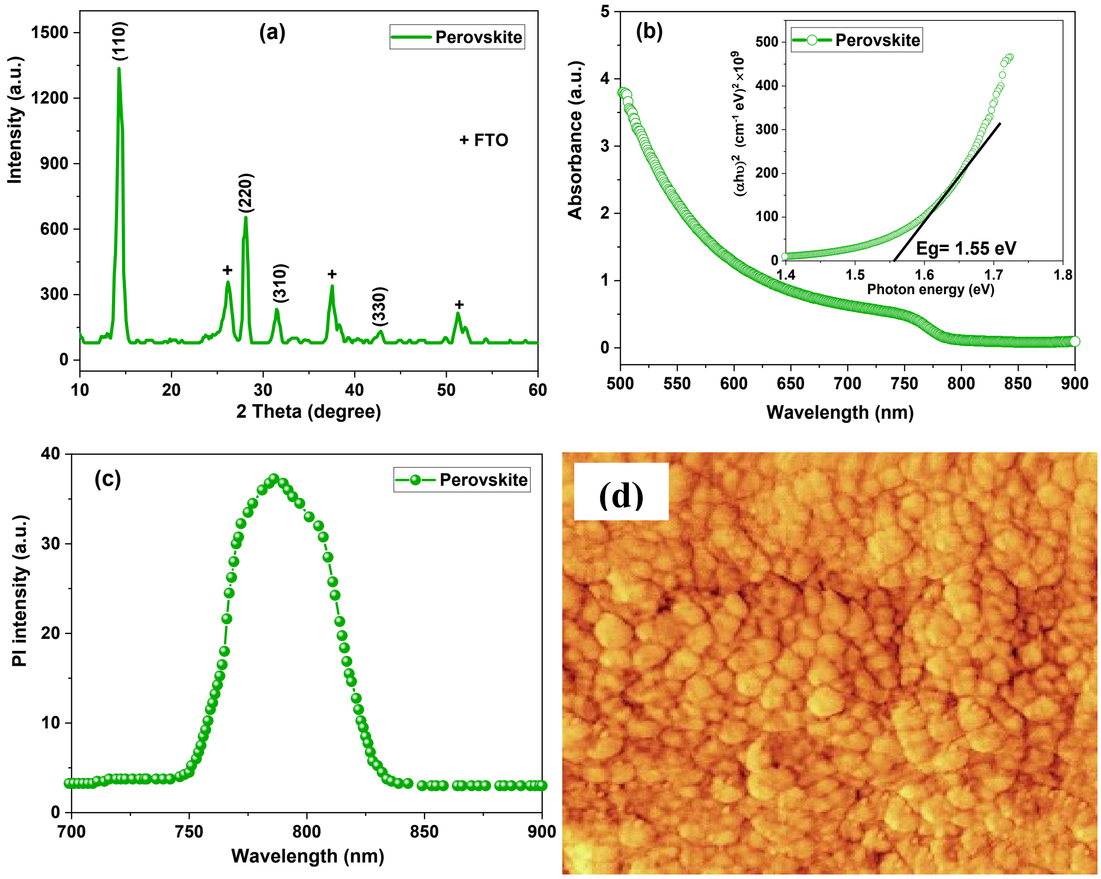

Figure 2 a) XRD pattern, b) absorption spectrum and Tauc plot (inset), c) steady-state PL, and d) AFM image for perovskite film.

2.3. ZnO-NPs measurements

Figure 3a illustrates the X-ray spectrum of the as-prepared Zn-NPs. In comparison with JCPDS reference, the diffraction peaks labeled as (100), (002), (101), (102), (110), (103), (200), (112), and (201) approved well with the crystalline structure of ZnO with the hexagonal wurtzite phase (JCPDS code: 36-1451) [5]. As depicted in the figure, the distinctive peaks for impurities are not revealed in the XRD spectrum, which emphasizes the preparation of ZnO-NPs with high purity. Table 1 displays the structural characteristics for the highest peaks showing in the XRD pattern of the as-prepared ZnO-NPs (100, 002 and 101). The Scherer equation was utilized to determine crystallite size [6]. The average size obtained for the ZnO-NPs was 39 nm. The obtained FTIR spectrum (Figure 3b) for ZnO-NPs exhibits a

wide absorption band in the low frequency region (400-500 cm^{-1}), which is related to the Zn-O stretching vibration of the ZnO structure. There are two vibration modes at 2872 and 3453cm^{-1}, which are attributed to the stretching vibration of asymmetric C-H and hydroxyl group (O-H), respectively. As shown in Figure 3c, EDS spectrum demonstrated that ZnO-NPs successfully synthesized without any impurities, which is consistent with XRD result. The morphology of the ZnO-NPs was analyzed via a FESEM image (Figure 3d). The image reveals semi-sphere nanocrystallites visible under the FESEM.

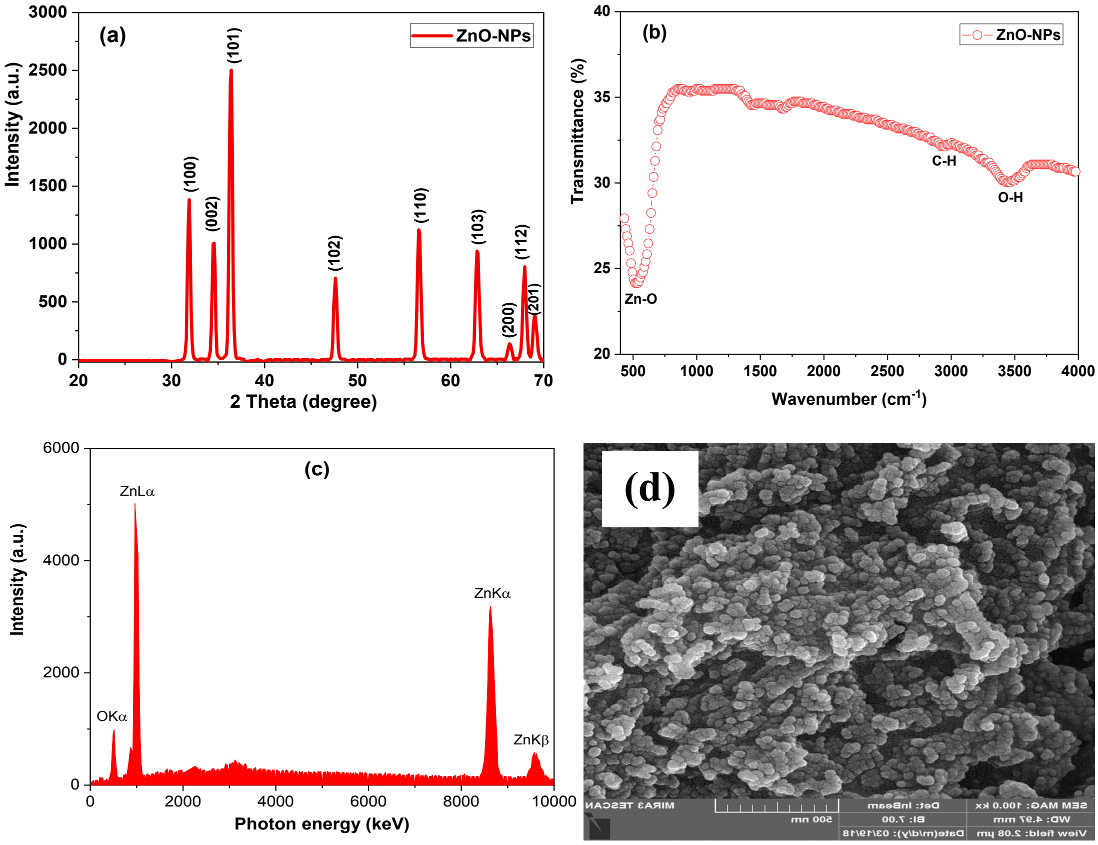

Figure 3 a) XRD pattern, b) FTIR spectrum, c) EDS spectrum, and d) FESEM image for ZnO-NPs.

Table 1. The microstructural properties of the as-prepared ZnO-NPs usedas ETM.

hkl[a]	Position (2θ°)	d-spacing[b] (nm)	FWHM[c]	Size (nm)
100	31.85	0.28	0.23	35.91
002	34.52	0.25	0.18	43.44
101	36.41	0.24	0.21	39.81

[a]*hkl*: Miller indices; [b] d: interlayer distance; [c] FWHM: full-width at half maximum

2.4. Device measurements

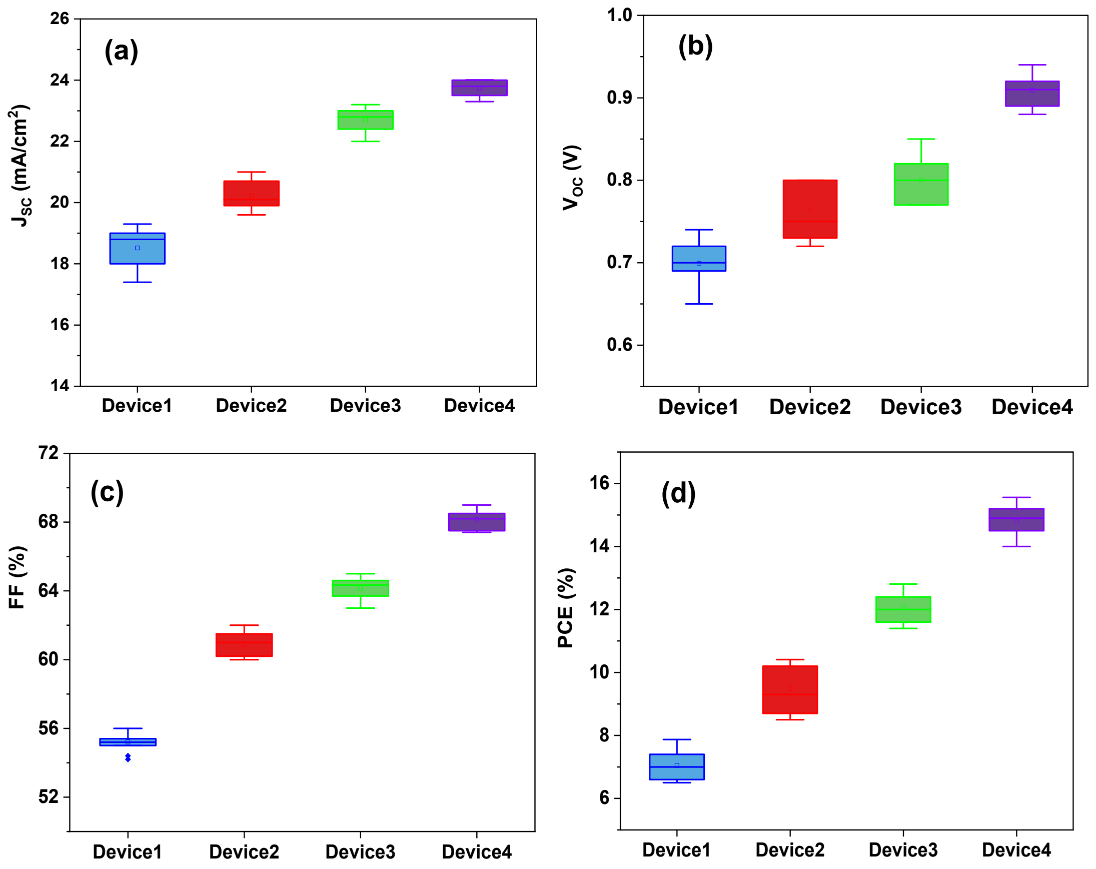

Figure 4. Device statistics of *a)* J_{SC}, *b)* V_{OC}, c) FF, and d) PCE of perovskite solar cells employing ZnO ETM. Every box exhibits the parameter distribution of 15 devices under the same operating environment.

References

1. M.K. A. Mohammed, *Inorganic and Nano-Metal Chemistry*, 2020, https://doi.org/10.1080/24701556.2020.1814336.
2. I.J. Raneen, M.K.A. Mohammed, *Optik*, 2016, 127, 6384-6389.
3. M.R. Mohammad, D.S. Ahmed, M.K.A. Mohammed, *J. Sol-Gel Sci. Technol.*, 2019, 90, 498–509.
4. P. Sakthivel, S. Foo, M. Thambidurai, P.C. Harikesh, N. Mathews, R. Yuvakkumar, G. Ravi, C. Dang, *J. Power Sources*, 2020, 471, 228443.

5. W. Yang, J. Wang, S. Luo, S. Yu, H. Huang, R. Sun, C.-P. Wong, *ACS Appl. Mater. Interfaces*, 2016, 8, 35545-35551.
6. D.S. Ahmed, M.K.A. Mohammed, *Chem. Pap.*, 2020, 74, 4033–4046.
